# Evidence of commitment to research partnerships? Results of two web reviews

**DOI:** 10.1186/s12961-019-0475-5

**Published:** 2019-07-30

**Authors:** Danielle de Moissac, Sarah Bowen, Ingrid Botting, Ian D. Graham, Martha MacLeod, Karen Harlos, Charity Maritim Songok, Monique Bohémier

**Affiliations:** 1 Faculty of Sciences, Université de Saint-Boniface, 200 ave de la Cathédrale, Winnipeg, MB R2H 0H7 Canada; 2 Applied Research and Evaluation Consultant, 322 Al Bennett Rd. RR3, Centreville, NS B0P 1J0 Canada; 3 Community Health Sciences, Rady Faculty of Health Sciences, University of Manitoba, 434-650 Main St, Winnipeg, Manitoba R3B1E2 Canada; 4 School of Epidemiology, Public Health and Preventive Medicine, School of Nursing, University of Ottawa, 451 Smyth, Ottawa, ON K1H 8M5 Canada; 5 Centre for Practice-Changing Research, The Ottawa Hospital Research Institute, 501 Smyth Road, Box 241, Ottawa, Ontario K1H 8L6 Canada; 6 School of Nursing, School of Health Sciences, UNBC Health Research Institute, University of Northern British Columbia, 3333 University Way, Prince George, BC V2N 4Z9 Canada; 7 Department of Business and Administration, Workplace Bullying and Mistreatment Partnership for Prevention (SSHRC), University of Winnipeg, 515 Portage Ave, Winnipeg, MB R3B 2E9 Canada; 8 Max Rady College of Medicine, University of Manitoba, Room S113 - 750 Bannatyne Avenue, Winnipeg, MB R3E 0W3 Canada; 9 Université de Saint-Boniface, 200 de la Cathédrale Avenue, Winnipeg, Manitoba R2H 0H7 Canada

**Keywords:** Health research, partnership research, research collaboration, Canadian health systems, integrated knowledge translation

## Abstract

**Background:**

Partnerships between academic researchers and health system leadership are often promoted by health research funding agencies as an important strategy in helping ensure that funded research is relevant and the results used. While potential benefits of such partnerships have been identified, there is limited guidance in the scientific literature for either healthcare organisations or researchers on how to select, build and manage effective research partnerships. Our main research objective was to explore the health system perspective on partnerships with researchers with a focus on issues related to the design and organisation of the health system and services. Two structured web reviews were conducted as one component of this larger study.

**Methods:**

Two separate structured web reviews were conducted using structured data extraction tools. The first review focused on sites of health research bodies and those providing information on health system management and knowledge translation (*n* = 38) to identify what guidance to support partnerships might be available on websites commonly accessed by health leaders and researchers. The second reviewed sites from all health ‘regions’ in Canada (*n* = 64) to determine what criteria and standards were currently used in guiding decisions to engage in research partnerships; phone follow-up ensured all relevant information was collected.

**Results:**

Absence of guidance on partnerships between research institutions and health system leaders was found. In the first review, absence of guidance on research partnerships and knowledge coproduction was striking and in contrast with coverage of other forms of collaboration such as patient/community engagement. In the second review, little evidence of criteria and standards regarding research partnerships was found. Difficulties in finding appropriate contact information for those responsible for research and obtaining a response were commonly experienced.

**Conclusion:**

Guidance related to health system partnerships with academic researchers is lacking on websites that should promote and support such collaborations. Health region websites provide little evidence of partnership criteria and often do not make contact information to research leaders within health systems readily available; this may hinder partnership development between health systems and academia.

**Electronic supplementary material:**

The online version of this article (10.1186/s12961-019-0475-5) contains supplementary material, which is available to authorized users.

## Introduction

The need for collaborative research approaches to address the many and complex problems currently facing the Canadian healthcare system is increasingly recognised [1, 2]. In addition to partnerships between researchers from diverse disciplines, intersectoral research teams, which include both researchers and various knowledge users, offer many benefits for improved health service delivery [3–5]. While some approaches to knowledge translation focus on transferring findings of research already completed, integrated knowledge translation approaches to research (like participatory research and engaged scholarship) engage researchers and knowledge users (patients, clinicians, local communities, policy-makers, or health system leaders and managers) in meaningful ways throughout the entire research process [6]. Many potential benefits of this approach have been reported for health systems and for the partners involved. Research partnerships may improve the quality of solutions [5] and enhance responsiveness of health services to community needs [7]. Greater research relevance and credibility may result in increased likelihood that research will be applied in practice [8] and have stronger impact [9]. Enhanced research capacity of both researchers and knowledge users [4], greater understanding of partners’ roles [9], personal and professional development [4], and enhanced skills and networks [3, 4, 9] may also result. Awareness of actual and potential benefits of such research partnerships has led to requirements by many major health research funders for greater participation by health system leaders in health research [10–13]. As a result, many researchers have sought research partners within the health system.

Recent research has found that, while health system leaders and managers often express support for the principles of collaborative research and articulate a number of potential advantages of such partnerships, there is limited guidance for healthcare organisations in how to select, build and manage effective research partnerships [14]. Conditions associated with successful partnerships have been identified [5, 8, 15–22], but research on this subject is often based on assumptions of researcher-driven initiatives [23] and fails to include health system leaders’ perspectives [24]. Very little is known about strategies for initiating and maintaining partnerships between health organisations and academic researchers to address issues of system organisation [25] – the focus of our study.

### Our study

“Building and Managing Effective Partnerships in Canadian Health Systems Research” (Ingrid Botting, P.I.) is a multi-phase, mixed-methods pan-Canadian study exploring how health systems partner with researchers relative to the design and organisation of the health system and health services [26]. As a first step in this research programme, the research team decided to identify current resources available to support research partnership development as well as to undertake a scan of how health regions across Canada were responding to increasing expectations of health system/academic partnerships. As an initial review of the scientific literature highlighted limited guidance in operationalising partnerships [14], the team decided to undertake a review of the grey literature to identify other potential resources available to support health systems in such research collaborations. This was followed by a review of health region public websites to determine what criteria, requirements or standards of practice were required of researchers for research partnerships to be considered. Ethics approval was received from the University of Manitoba Health Research Ethics Board.

## Methods

Content analysis of two website types was conducted. Research team members, all with integrated knowledge translation expertise in health services, provided oversight of all phases of the study. Activities were conducted between November 2017 and April 2018. Each of the two web reviews is described separately below.

### Web review 1 (Health system support)

This review aimed to identify resources or guidance for research partnerships published on websites believed by the research team to be those most likely to be accessed by either health leaders or researchers interested in research partnerships. Additional sites were included as they were identified. These sites included Canadian and well-known international websites that (1) address issues of health system organisation and functioning, (2) are home to Canadian research funding bodies or (3) are sponsored by organisations promoting knowledge translation or evidence use in healthcare.

Inclusion criteria for the resources selected within these sites was that they addressed both health system change/health service organisation and the practical issues of ‘how to’ establish or manage health system/academic partnerships. Exclusion criteria were resources that (1) were limited to clinical research, researcher relationships with communities or individuals, or collaboration only among academic researchers; (2) were limited to end-of-project (knowledge transfer) activities; or (3) addressed only knowledge user capacity-building or assessment of research capacity. All pages of each site were reviewed; initial selection of potential resources was done by title or description. Resources linked to these sites were also reviewed. All applicable resources were assessed according to a standardised template reflecting principles for effective research partnerships identified through the literature and such items included recognition of stages and diversity of partnerships, recognition of challenges in initiating and maintaining research partnerships, and presence of specific guidance for health systems in establishing and promoting effective research team work (see Additional file 1).

### Web review 2 (Canadian health regions)

This second review explored websites of all Canadian health regions (*n* = 64). These structures, which include regional health authorities, provincial health delivery bodies, local health integration networks and the Integrated Health and Social Service Centres (CISSS) and Integrated University Health and Social Service Centres (CIUSSS) of Quebec, are diverse in structure and size, and reflect the provincial responsibility for health service provision found in Canada. Sites specific to hospital or health centres were excluded as the focus was on regional organisation. This review focused on (1) evidence of research partnerships by health regions; (2) messages targeted to potential research collaborators; (3) extent to which regional review processes demonstrate requirements for partnership, offer guidance or address partnership issues; and (4) accessibility of information relevant to research partnerships for academic researchers (i.e. contact information for research personnel).

All pages of each site were reviewed using a standard template exploring and documenting the following elements: stated commitment to research and interest in collaborating with academic researchers; evidence of research collaboration; evidence of guidance for research partnerships; and overall messaging related to the importance of research, evidence use and knowledge translation activities (see Additional file 2). Availability of research support services, as well as information and submission forms pertaining to research review processes such as impact and access assessment, ethical review within organisations and formal approval by a Research Ethics Board, were noted.

As it was recognised that regions may be using resources not linked to the public websites, key research contact information was retrieved; email or telephone follow-up to each region was conducted to confirm that all relevant publicly available information had been collected pertaining to research collaboration and guidance to support research partnerships.

### Analysis

Directed content analysis [27] was used to review, analyse and synthesise website content [28]. This method was chosen as it is unobtrusive, context sensitive, simple and economical [28]. This research method uses prior research as guidance for developing initial codes, which are revised and refined as analysis proceeds. A systematic classification process was conducted by coding; themes and patterns were also identified. Separate structured extraction templates, based on evidence of factors related to effective partnerships [14, 29–31], were developed by team members for each of the two reviews.

The templates developed for review served as the data extraction tools for each web review. Qualitative analysis was conducted using a deductive approach [32]. Analysis and synthesis of data sources for the first review was conducted by one researcher; other team members undertook a second review of select sites. Analysis and synthesis for the second review was conducted by two researchers, in consultation with the research team members. A summary matrix was created for regional data collected to facilitate synthesis and quantification of data collected through the review. The process of contacting individuals responsible for research was also documented. When direct contact information was not provided, staff directories, online searches or general enquiry phone numbers or email addresses were used. Up to two follow-up attempts were made after the initial request for information. Frequencies and percentages, calculated with Excel (Microsoft 2010), were used to report quantitative findings.

## Results

### Web review 1 (Health system support)

#### Limited resources to guide research partnerships

While many websites referred to partners, collaborations or sponsors, few resources providing guidance for effective health system-research partnerships were found; most of those found provided only minimal guidance and were not developed for use by health leaders.

In total, 38 websites were assessed; from these, 46 potential resources guiding partnerships were identified. Of these, only 12 met criteria to merit a full review and only 10 provided concrete guidance. None was written specifically for health system leaders; however, 4 were written for a general or unspecified audience. Most audiences identified were researchers [6], policy-makers [3] or other audiences [2]. Seven noted that research may be health system initiated; however, no guidance was given. Most [9] provided some suggestions for establishing partnerships, but few of these were comprehensive and only two gave more than a few suggestions for promoting effective teams. Four focused on international development work. Two were short, recent blog posts. Only one resource directly addressed the topic of our research.

Most resources (such as webinars, courses or tools) intended for knowledge users focused on developing research-related skills of health personnel; few addressed relationship building or the skills needed to establish meaningful partnerships or influence research agendas.

#### Absence of resources for knowledge coproduction

The concept of knowledge translation (including terms such as knowledge transfer, knowledge to action or knowledge mobilisation) was found frequently in this review. Some useful links were provided to knowledge translation theory. However, knowledge transfer (referring to strategies to communicate research results on research already completed) remained the dominant approach found in resources reviewed. Resources to support knowledge coproduction were conspicuous by their absence.

### Web review 2 (Canadian health regions)

#### Limited evidence of research partnerships

Overall, there were limited references to research collaborations or to a role in research in the review of health region sites. Generally, there was limited mention of, or reference to, either research or commitment to evidence-informed system design or organisation. Of the 64 health region websites, 38 (59.4%) made no mention of research on the front page. Few mission or vision statements mentioned a commitment to knowledge-to-action strategies (i.e. knowledge transfer, knowledge translation/mobilisation) (*n* = 16, 25%), evidence (*n* = 17, 26.5%) or research (*n* = 7, 10.9%).

Involvement in research collaborations was noted on approximately half the websites (*n* = 31, 48.4%), although specific examples generally referred to research of a clinical, basic science or community/population health nature. Formal affiliation to universities or research institutes was more evident in Quebec websites, as were examples of research collaborations. As might be expected, rural and remote health regions across Canada were the least likely to address research collaboration with academic researchers. These regions were more likely to provide information on community engagement, and regions with a large indigenous population were most likely to provide links to and emphasis on using specific ethical guidelines for research with indigenous peoples.

There was a striking absence of evidence in this web review of (1) stated requirement for partnership, (2) criteria for engaging in health organisation research partnerships and (3) assessment of collaboration between health regions and researchers. Only two of the 64 regions (3.1%) provided any evidence of guidance around research partnerships above standard review requirements, although many did state a requirement for organisational or programme approval. Less than one quarter (*n* = 15, 23.4%) provided website information related to research support or facilitation services offered by the region. Information of research review processes was readily accessible on 39.1% of websites (*n* = 25). The majority of sites of large- and medium-sized health regions provided research review contact information or guidelines and/or forms for ethics, research and access reviews; information on research review processes and application forms, where available, were remarkably similar.

#### Limited accessibility of health regions to researchers

Challenges were experienced in both identifying and contacting individuals responsible for research in the majority of health regions. Responsibility for research was often unclear and direct research-related contact information was often not provided (*n* = 45, 70.3%). Attempts to connect with the identified person on the website were successful in less than 30% of cases. Median wait time for response was prolonged (18 days). In 25% of cases, no response was received, even after a minimum of three attempts at making contact with the individual responsible for research (in some cases, these attempts were in addition to contacts made to identify this individual) over a 10-week period. Email appeared to be more effective than telephone contact. Proceeding through a general phone number or website form was least successful – individuals answering often did not know who to refer to for research questions and some did not respond at all. Where the only contact was an ethics or research review committee, delays in response to requests for contact were also common.

## Discussion

These two web reviews were undertaken as preliminary activities to inform a larger programme of research designed to explore health system perspectives on research partnerships with academia on topics related to health system organisation. Some of the results reported here were unexpected and provide insight into potential barriers to such collaborations. Consistent with preliminary findings of a scientific literature review [14], few resources or guidance in how to establish and successfully manage research partnerships are made available through health system support websites. Furthermore, an absence of evidence of research partnerships with a specific focus on health system design and organisation and criteria required by researchers to collaborate with health systems was observed in the review of Canadian health region websites. While a number of authors have acknowledged similar challenges in initiating and implementing multi-sectoral actions [33, 34], there appears to be less attention given to health system and research partnerships. The absence of specific guidance and concrete examples of successful collaborations focused on health system organisation suggests that effective research partnerships may not be currently viewed as a priority by health organisations.

While recent research stresses the importance of entering partnerships with health leaders early on in the process to improve research relevance and use [15, 18, 22], the majority of resources identified on health system support websites focused simply on dissemination of research at the end of the study (knowledge transfer or end-of-project knowledge translation) rather than on coproduction of research reflecting user concerns. This suggests that research findings may not have been integrated into materials currently available. In addition, most resources were directed to researchers or ‘knowledge translation’ practitioners rather than to health system leadership.

There does, however, appear to be some indication that change may be occurring, and that more attention to ‘coproduction’ may be emerging. For example, leading international organisations, such as the WHO Alliance for Health Care Policy and Systems Research [35], are providing consistent aspirational statements about the importance of meaningful collaboration between the health system and researchers while appearing to move away from an emphasis on simple, one-way research knowledge transfer. Such documents and dialogue that these efforts spur may provide a needed foundation for practical steps to support health system leaders in initiating and managing research partnerships.

Recognising that public websites of health regions may not post all relevant information either in report form or links to guidelines, we attempted to confirm, with appropriate contacts from each region, whether additional materials were used. However, these follow-up requests did not result in additional materials being found with respect to either evidence of research partnerships or processes supporting such collaborations. Furthermore, contact with personnel within the health system with responsibility for research and/or relationships with academic researchers proved challenging. The difficulty in identifying and making contact with these individuals suggests that academic researchers may face significant barriers in identifying and establishing relationships with potential health system partners.

The observation that academic/health system partnerships appeared to be more apparent in Quebec may reflect the fact that, following the systemic integration of health and social services in 2015, Integrated University Health and Social Service Centres were created, improving visibility of the academic role in both research and education within those health regions. However, further research is required to determine whether these formal academic affiliations have led to greater research collaboration, or whether such affiliations have been more receptive to system-prioritised research.

In general, absence of information on research/health system partnerships in both reviews was in clear contrast to the presence of general information and specific guidance and information for health systems to engage in partnership with a number of other key stakeholder groups. More information and guidance was found on community engagement, particularly for engaging with indigenous communities (e.g. Canadian Institute of Health Research 2013) [36]; patient engagement (e.g. Canadian Foundation for Healthcare Improvement 2018) [37]; and research and policy relationships in an international development context (e.g. Canadian Coalition for Global Health Research 2017) [38] as well as professional, inter-professional and inter-organisational collaboration (e.g. Agence de la santé et des services sociaux de l’Abitibi-Témiscamingue 2010) [39]. Reasons for this disparity in coverage require further investigation.

A limitation to the review of health region websites is that content of publicly accessible messages provided on health region websites may not accurately reflect all initiatives or resources. However, as previously noted, follow-up requests for information made to research personnel in these regions confirmed the absence of specific guidelines for partnerships in most regions. This limitation does not apply to the first (health system support) review as the websites analysed would be those most likely to be consulted by health leaders and researchers. Another limitation of both reviews is that frequency of website updates and redesign means that results of such reviews could vary on a monthly basis. Monitoring of several sites since the reviews were completed indicates, however, that the observed trends are still apparent.

## Conclusion

This research, consisting of two focused web reviews, found an absence of identifiable guidance related to health system/academic research partnerships in websites that could be expected to provide resources and support for such collaborations. This finding does not reflect the priority given to research partnerships by many health research funding agencies. Canadian health region websites provide little evidence not only of research partnerships pertaining to health system organisation but also of research partnership criteria in contrast to attention directed towards other forms of partnerships. Difficulties in determining research contacts within health regions are common and may present challenges in the formation of research collaborations. Further research is needed to gain insight into whether Canadian health leaders partner with academia and which strategies are used to foster such partnerships.

## Additional files

Additional file 1: Extraction table for review of web resources for health systems. (DOCX 508 kb)

Additional file 2: Extraction table for review of Canadian health regions’ websites. (DOCX 497 kb)

## Data Availability

A list of all websites accessed for web review 1 (Health system support) could be made available; however, the rapidly evolving nature of websites would not necessarily enable replication of what resources were posted. A dataset was created for web review 2 (Canadian Health Regions); however, for confidentiality and ethical considerations, we are unable to make this dataset available as it includes names of informants and specific regions. Furthermore, some health regions have changed (for example, amalgamation in Saskatchewan) since the review was undertaken; results could therefore not necessarily be replicated in all regions. Copies of the data extraction sheets for both reviews are provided in the additional files.
