# Supplementary material for: Evidence of commitment to research partnerships? Results of two web reviews
Source: Health Res Policy Syst. 2019 Jul 30;17:73. doi: 10.1186/s12961-019-0475-5 (PMC6668137; doi:10.1186/s12961-019-0475-5)
Supplement: Supplementary file 1 — Extraction table for review of web resources for health systems. (DOCX 508 kb) [file 12961_2019_475_MOESM1_ESM.docx]

| **Name of Resource Reviewed** |  | |
| --- | --- | --- |
| **Date of resource** |  | |
| **Authors** |  | |
| **Location of Resource** |  | |
| **Criteria** | **Interpretation** | **Comments** |
| Provides guidance on research partnerships | General assessment of resource  Level 0: Identifies importance of research partnerships. If level 0, do not complete rest of tool.  Level 1: Provides specific guidance |  |
| Primary Audience | Note if audience is:   - unclear, unspecified - researcher only - KU only - directed to both researchers and KUs - Whether addresses clinicians, policy makers, managers, community groups |  |
| Recognizes culture of decision-making | Level 1. Refers to cultural differences between DMs and academia  Level 2. Provides strategies for addressing cultural differences |  |
| Recognizes diversity of research partnerships | Level 1. Recognizes various types of research partnerships  Level 2. Provides strategies relevant to different types of partnerships |  |
| Recognizes different stages of partnerships | Level 1. Recognizes various stages of research partnerships  Level 2. Provides strategies relevant to specific stage of partnerships between DMs and academics |  |
| Addresses common challenges in partnership | Level 1. Recognizes challenges  Level 2. Provides strategies for addressing challenges of (note challenge identified):   - time - time frames - communication - power - cultural differences - funding requirements - facilitation/networking skills - diverse agendas |  |
| Strategies for health system initiation | Level 1. Recognizes potential differences between researcher and health system initiated research  Level 2. a. Provides guidance on promoting research on DM priorities |  |
| **ESTABLISHING PARTNERSHIPS** | | |
| Provides practical guidance on issues related to establishing partnerships   - Identifying skilled researchers - Advisory structures - Early involvement - Strategies for developing shared agenda/priorities - Cost sharing - Communication strategies within org - Communication strategies within team - Clear contracts - Relationship broker role - Appropriate resourcing for development - Selection of KU participants - Clear roles and responsibilities of KU team participants - Guidelines for team member selection | Level 1. Recognizes issues that may need to be addressed for in establishing effective partnerships  Level 2. Provides generic guidance on topics listed (list specific topics covered)  Level 3. Provides guidance specific to health system change/health service organization |  |
| **PROPOSAL DEVELOPMENT** | | |
| Provide guidance for identifying questions both important and researchable | Guidance is provided. Yes/No |  |
| Provides guidance on funding opportunities | Yes/No |  |
| Provides guidance on roles of partners | Yes/No |  |
| Makes link to project management principles | Yes/No |  |
| Provides practical guidance for supporting role of organizational team members | Yes/No |  |
| **PROMOTING EFFECTIVE TEAMS** | | |
| Provides guidance on how to create respectful environments | Yes/No |  |
| Encourages opportunities for face-to-face meetings | Yes/No |  |
| Identifies need for adequate time for team development | Level 1: Recognizes need  Level 2: Suggests strategies |  |
| Provides clear guidance on team orientation | Level 1: Identifies need  Level 2: Provides guidance on topics to be addressed |  |
| Provides guidance on developing agreements around rules of engagement | Level 1: identifies need  Level 2: provides specific guidance on strategies, topics |  |
| **PROMOTING APPROPRIATE/DISSEMINATION/ IMPLEMENTATION** | | |
| Promotes early meaningful involvement of planned user | Level 1: stresses importance  Level 2: suggests specific strategies |  |
| Ensure KU reps have credibility/time | Level 1: stress importance  Level 2: provides specific suggestions |  |
| Undertake early planning for dissemination and action | Level 1: identifies need  Level 2: provides specific guidance |  |
| Importance of allocation of resources for dissemination and implementation planning | Level 1: Identifies need  Level 2: Provides specific suggestions |  |
