# Supplementary material for: Evidence of commitment to research partnerships? Results of two web reviews
Source: Health Res Policy Syst. 2019 Jul 30;17:73. doi: 10.1186/s12961-019-0475-5 (PMC6668137; doi:10.1186/s12961-019-0475-5)
Supplement: Supplementary file 2 — Extraction table for review of Canadian health regions’ websites. (DOCX 497 kb) [file 12961_2019_475_MOESM2_ESM.docx]

Additional file 2: Appendix B - Extraction table for Review of Canadian Health Regions’ Websites

**NAME OF REGION:**

**WEBSITE:**

| **Criteria** | **Addressed directly** | **Partially**  **addressed or implied** | **Not**  **addressed** | **Comments** |
| --- | --- | --- | --- | --- |
| Importance of/commitment to role in research noted |  |  |  |  |
| Concept of partnership/collaboration between health system and academics noted |  |  |  |  |
| Clear statement of interest in partnering with academic researchers |  |  |  |  |
| Internal R and E unit |  |  |  |  |
| Contact person for research partnership questions clearly identified |  |  |  |  |
| Guidelines or criteria for partnership stated |  |  |  |  |
| Commitment to evidence informed practice/policy stated |  |  |  |  |
| Approach to KT |  |  |  |  |
| Clear statement of collaboration in area of systems, organizations of services |  |  |  |  |
| Examples of research partnership provided |  |  |  |  |
| - general |  |  |  |  |
| - clinical |  |  |  |  |
| - program specific |  |  |  |  |
| - system design |  |  |  |  |
| Research Review/Impact and Access Committee process and guidelines readily accessible |  |  |  |  |
| Impact and Access guidelines require partnership with health region staff at levels appropriate for the research question |  |  |  |  |
| Evidence of knowledge exchange opportunities, meetings with researchers |  |  |  |  |
| Evidence of formal research relationships with academic centres |  |  |  |  |

**GENERAL OBSERVATIONS:**
